# Nebulized milk exosomes loaded with siTGF-β1 ameliorate pulmonary fibrosis by inhibiting EMT pathway and enhancing collagen permeability

**DOI:** 10.1186/s12951-024-02721-z

**Published:** 2024-07-23

**Authors:** Chong Qiu, Zhenyu Zhao, Chenglin Xu, Ranran Yuan, Yuxuan Ha, Qingchao Tu, Houqian Zhang, Zhen Mu, Quanlin Xin, Yu Tian, Aiping Wang, Hongbo Wang, Yanan Shi

**Affiliations:** 1 School of Pharmacy, Key Laboratory of Molecular Pharmacology and Drug Evaluation (Yantai University), Collaborative Innovation Center of Advanced Drug Delivery System and Biotech Drugs in Universities of Shandong, Ministry of Education, Yantai University, Yantai, 264005 PR China; 2 College of Life Science, Yantai University, Yantai, 264005 P.R. China; 3 Ontario Virtual School, 4789 Yonge Street, Unit 705, Toronto, ON M2N 0G3 Canada; 4 State Key Laboratory for Quality Ensurance and Sustainable Use of Dao-di Herbs, Artemisinin Research Center, Institute of Chinese Materia Medica, China Academy of Chinese Medical Sciences, Beijing, 100700 China

**Keywords:** siRNA delivery, Milk exosomes, Pulmonary fibrosis, Epithelial mesenchymal transition, Collagen permeability

## Abstract

Pulmonary Fibrosis (PF) is a fatal disease in the interstitial lung associated with high mortality, morbidity, and poor prognosis. Transforming growth factor-β1 (TGF-β1) is a fibroblast-activating protein that promotes fibrous diseases. Herein, an inhalable system was first developed using milk exosomes (M-Exos) encapsulating siRNA against TGF-β1 (MsiTGF-β1), and their therapeutic potential for bleomycin (BLM)-induced PF was investigated. M-siTGF-β1 was introduced into the lungs of mice with PF through nebulization. The collagen penetration effect and lysosomal escape ability were verified in vitro. Inhaled MsiTGF-β1 notably alleviated inflammatory infiltration, attenuated extracellular matrix (ECM) deposition, and increased the survival rate of PF mice by 4.7-fold. M-siTGF-β1 protected lung tissue from BLM toxicity by efficiently delivering specific siRNA to the lungs, leading to TGF-β1 mRNA silencing and epithelial mesenchymal transition pathway inhibition. Therefore, M-siTGF-β1 offers a promising avenue for therapeutic intervention in fibrosis-related disorders.

**Supplementary Information:**

The online version contains supplementary material available at 10.1186/s12951-024-02721-z.

## Graphical Abstract

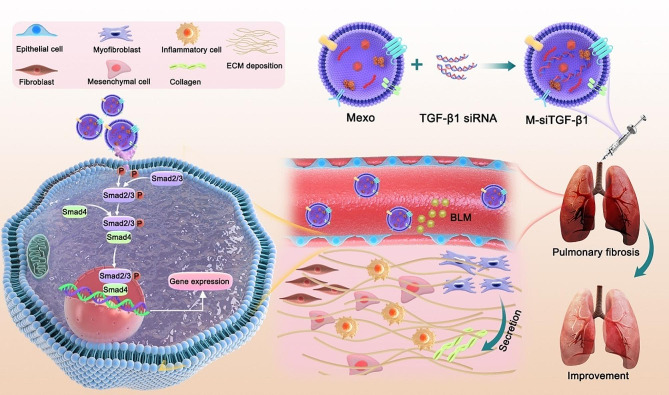

## Introduction

Pulmonary fibrosis (PF) is a relentless interstitial lung disorder marked by chronic progression and a high fatality rate, primarily due to respiratory failure, with affected individuals typically surviving only 3 to 5 years after being clinically diagnosed [1, 2]. The incidence of pulmonary fibrosis is increasing over the years due to factors such as occupational exposure, environmental pollution, and viral infection [3–6]. Although currently marketed drugs, pirfenidone and nintedanib, partially alleviate clinical symptoms, they fail to improve survival rates [7, 8]. While lung transplantation is the most efficacious intervention for PF, its utility is hampered by the criticality of referral timing and diminished post-transplant survival rates [9, 10]. Therefore, a deeper investigation into the pathogenesis of PF and the identification of innovative therapeutic targets are critically significant from a clinical perspective.

Although the underlying mechanisms of PF remain complex and elusive, it is generally believed that persistent injury to the alveolar epithelium plays a central role in the disease’s development and progression [11, 12]. Damaged lung epithelial cells initiate epithelial mesenchymal transition (EMT), causing sustained activation of mesenchymal cells and participation in the process of matrix remodeling, resulting in excessive deposition of extracellular matrix (ECM) and the formation of fibrous scar tissue, ultimately leading to lung failure [13, 14]. Prior research has shown that the initiation of TGFβ signaling involves TGF-β binding to its type II receptor, which then activates the type I receptor endowed with a kinase domain. This domain phosphorylates Smad2/3, leading these phosphorylated Smads to associate with Smad4, forming a heterotrimeric complex that translocates to the nucleus [15, 16]. After its translocation to the nucleus, the heterotrimeric complex modulates the expression of a variety of genes including connective tissue growth factor (CTGF), α-smooth muscle actin (α-SMA), and collagen, through direct binding to their promoters [17–19]. These results underscore the viability of targeting TGF-β1 in developing therapeutic strategies for pulmonary fibrosis. Small interfering RNA (siRNA) has surfaced as a pioneering class of therapeutics [20]. Specifically, siRNAs that target TGF-β1 have shown potential as therapeutic agents in mitigating fibrosis in the heart and kidneys [21–23]. In the same context, we hypothesized that pulmonary fibrosis could be controlled by downregulating TGF-β1. The biggest limitation of gene interference therapy when applied to in vivo treatment is the lack of efficient nucleic acid drug delivery systems [24, 25]. Lipid- and polymer-based systems improve the stability and delivery efficiency of siRNAs by protecting them from degradation [26, 27]. However, these synthetic systems have some disadvantages such as toxicity, nonspecific uptake, and immunogenic effects [28]. Therefore, the identification of effective and biocompatible vectors to overcome the issues of targeted siRNA deletion.

Exosomes, which are extracellular vesicles with diameters ranging from 30 to 150 nm, are ubiquitously present in numerous cell types and are crucial for cell-to-cell communication [29, 30]. Their distinctive characteristics, such as targeted tissue delivery, extended half-life, high biocompatibility, and low toxicity, establish them as ideal platforms for drug delivery applications [31–34]. However, the cumbersome process of cell culture expansion and the inherently low exosome yield limit the clinical translatability of these systems [35]. Recognizing the limitations in traditional sources, our research identifies milk as a viable alternative for exosome procurement, offering a potential avenue for drug delivery platforms. Previous studies have shown that encapsulation of siRNA into milk exosomes (M-Exos) improves its gene silencing capabilities in HEK293 cells [36]. Sadri et al. reported that MExos could overcome the placental barrier [37]. In another study, M-Exos in transporting synthetic miRNAs resulted in altered placental gene expression and increased fetal implantation in mice [38].

In this study, we utilized M-Exos loaded with TGF-β1 siRNA to inhibit EMT, a pivotal process in the progression of PF. We hypothesized that M-siTGF-β1 could effectively dampen the TGF-β1-mediated activation of the Smad2/3 signaling pathway, thereby mitigating EMT in pulmonary fibrosis. To validate our hypothesis, we executed a series of proof-of-concept, utilizing a respirable nanoparticle-mediated RNA interference strategy to specifically target TGF-β1. This approach aimed to curtail the advancement of PF in a BLM-induced pulmonary fibrosis mouse model, as depicted in Graphical abstract.

## Materials and methods

### Isolation and characterization of M-Exos

Exosomes were isolated from raw milk by differential centrifugation techniques in accordance with previously established protocols [39, 40]. Briefly, fresh milk underwent centrifugation at 5,000 × g for 20 min at 4 °C, followed by a second centrifugation at 12,000 × g for 60 min at 4 °C to eliminate the remaining fat and debris. Then centrifuged (70,000 × g; 30 min; 4 °C) to remove the casein. The resulting whey was filtered through 0.22-µm membranes and subjected to ultracentrifugation (100,000 × g; 90 min; 4 °C) using an ultracentrifuge (Sorvall WX100+, Thermo, MA). The obtained precipitate was purified by centrifugation. The morphology of the exosomes was examined using transmission electron microscopy (JEM-1230; JEOL, Tokyo, Japan). Diameter and particle number of M-Exos were determined using a nanoparticle tracking analyzer (NTA, Malvern Panalytical, UK), and protein quantification was performed using the BCA protein assay kit (Cat. No. P0010, Beyotime, China). The exosome signature proteins CD63 (1:1000, Ab134045, Abcam, UK), ALIX (1:1000, ab275377, Abcam) and Tsg101 (1:1000, Ab125011, Abcam) were was confirmed through western blot analysis. Additionally, the stability of M-Exos was assessed by NTA after a 48 h incubation at 37 °C in a slightly acidic environment, which simulates lung conditions (PBS with 10% Exosome-Depleted FBS One Shot ^TM^ (Gibco, A27208-03), pH 7.4 or 6.8) [41].

### Preparation and identification of siRNA loaded-M-Exos

To introduce the siNC (Forward: 5’- UUCUCCGAACGUGUCACGUTT-3’, Reverse: 5’-ACGUGACACGUUCGGAGAATT-3’) and siTGF-β1 (Forward: 5’- CCCAAGGGCUACCAUGCCAACUUCU-3’, Reverse: 5’-AGAAGUUGGCAUGGUAGCCCUUGGG-3’) into M-Exo, electroporation, ultrasonic method and the modified CaCl_2_ method were conducted. For electroporation, 100 µg M-Exos were proportionally mixed with 200 pmol siRNA in 500 µL of PBS solution, and subjected to electroporated at 220 V, 10-ms pulse three times with an interval of 2 s using Gene Pulser Xcell™ (BIO-RAD, USA) [42]. For ultrasonic method, M-Exos and siRNA were mixed at a 1:5 (mass/mass) ratio in PBS. The on/off cycle was performed 6 times for 30 s each of 30 W, with a 2-minute cooling period between cycles. The sample was incubated at 37 °C for 30 min after sonication (SCIENTZIID, Ningbo Scientz Biotechnology Co., Ltd.) [43–45]. For the modified CaCl_2_ method, 200 pmol siRNA and 100 µg M-Exos were mixed in PBS, followed by the addition of CaCl_2_ (100 mM). The mixture was then chilled on ice for 30 min. it underwent a heat shock at 42 °C for 60 s and was subsequently cooled on ice for 5 min [42, 46, 47]. The intratracheal quantitative drug delivery device was purchased from Shanghai Yuyan Scientific Instrument Co. Ltd and mainly consisted of a nebulizing jet head and a high-pressure syringe. The morphology and diameter of M-siTGF-β1 were verified by transmission electron microscope and NTA.

The encapsulation efficiency (EE%) was determined using the Quant-iT™ RiboGreen™ RNA Assay Kit (R11490, Invitrogen, USA). Briefly, M-Exo with siRNA is prepared and nebulized as described above and the total and free RNA content of the sample is measured. Total RNA is obtained by “breaking the emulsion” of the exosome sample with an equal volume of 2% TritonX100 solution, and the fluorescence intensity is measured using an enzyme marker with excitation/emission at 480/520 nm.

### Cell culture

Bronchial epithelium (BEAS-2B) cells and human fetal lung fibroblast1 (HFL1) cells, acquired from the Chinese Academy of Sciences (Shanghai, China), were cultured under different conditions: BEAS-2B cells were propagated in DMEM with 10% FBS and 1% penicillin-streptomycin; HFL1 cells were cultured in Ham’s F-12 K Medium with 10% FBS, 1% Glutamax, 1% non-essential amino acids, and 1% sodium pyruvate, similarly at 37 °C and 5% CO_2_.

### Establishment of BLM-induced cell damage model and siRNA loaded-M-Exos intervention

BEAS-2B cells were subjected to BLM exposure (1 µg/mL) (Maokang Biotechnology Co., Ltd, Shanghai, China) for 24 h when the confluence reaches 80%. Following BLM treatment, the cells underwent subsequent treatment with M-Exo / naked siTGF-β1 / siNC loaded- M-Exo (M-siNC) / siTGF-β1 loaded-M-Exo (M-siTGF-β1) or PBS as the control group for next 24 h.

### Collagen barrier penetration assay in vitro

HFL1 cells were inoculated in 24-well plates at 8 × 10^4^ cells/well and cultured for 24 h. BLM (1 µg/mL) was added to induce HFL1 to form a collagen barrier. After another 24 h, the existing culture medium was replaced with fluorescently labeled M-Exo-DiR, FAMsiRNA and M-Exo-FAMsiRNA respectively. The cells were incubated in darkness at 37 °C for varying durations of 6 h, 12 h and 24 h. Fixed with 500 µL 4% paraformaldehyde in each well. DAPI reagent (100 µL) was used for nuclear staining. High-resolution imaging of the treated cells was performed using a live cell imaging system (Cytation, BioTek™, USA).

DiR was diluted 5-fold with 1×PBS to prepare a dye working solution at a concentration of 100 µM. The dye working solution was added to the bovine milk exosome suspension, mixed in a 1.5 mL centrifuge tube, mixed by ultrasonication for 1 min, and then incubated at 37 °C for 30 min. After the reaction was terminated by placing the tube in a refrigerator at 4 °C, the liquid was transferred to an ultrafiltration centrifuge tube (30 kD), and then centrifuged at 3,500 rpm for 10 min. The upper layer of the liquid was rinsed several times with PBS and then centrifuged to give M-Exo-DiR.

### Wound-healing assay

BEAS-2B cells were inoculated in growth medium at 1 × 10^5^ cells/well in 24-well plates and treated with BLM for 24 h. Cells were incubated with medium containing M-Exo /naked siTGF-β1 / M-siNC / M-siTGF-β1 and PBS, respectively, for another 24 h. The monolayer cell was scraped using pipette tips, washed thrice with PBS. The migration of the cells was monitored with the microscope for 0 h, 12 h and 24 h after wounding.

### Lysosomal escape

BEAS-2B cells were inoculated in 24-well plates at 8 × 10^4^ cells/well and cultured for 24 h. Cy5-siRNA (Gene Pharma Co. Ltd, Shanghai, China) labelled-Exos were incubated with BEAS-2B cells. After a certain time, unabsorbed Exos were washed off with PBS. Incubated with lysotracker green fluorescent probe (200 µL) at 37 °C for 30 min to stain the lysosomes, stained with Hochest 33,342 for nucleus visualization [48]. After a final wash, cells were imaged using a multifunctional cellular microwell imager.

### Animals

Male C57BL/6 mice, aged between eight to ten weeks, were procured from Jinan Pengyue Experimental Animal Breeding Co., Ltd. (Jinan, China). These specific pathogen-free (SPF) grade animals were housed individually in cages, with unrestricted access to food and water. The vivarium maintained a 12-hour light-dark cycle, ensuring an ambient temperature of (22 ± 2) °C and a relative humidity of (50 ± 10) %. All procedures involving these animals were conducted in strict accordance with the ethical standards and guidelines set forth by the Yantai University Animal Care and Use Committee.

### In vivo tracking of M-Exo

DiR-stained exosomes (DiR-M-Exo) were nebulized into C57BL/6 mice through the trachea. In vivo imaging sessions were conducted at multiple time points post-inhalation, specifically at 0 h, 4 h, 1 d, 3 d, 5 d, and 7 d, utilizing a Small Animal Imaging System (IVISKinetic, USA). In vitro fluorescence imaging of mouse organs was performed to observe the retention of DiR-M-Exo in various tissues [49].

### Animal treatment with BLM and M-siTGF-β1

C57BL/6 male mice, were anaesthetized on day 0 and treated with 1.5 mg/kg BLM solution (50 µL/mouse) via endotracheal nebulization of BLM dissolved in saline, and control mice were treated with equal volume of normal saline. Fourteen days post-BLM exposure, the mice were randomly allocated into six distinct groups, nebulized with normal saline, M-Exo, Naked siTGF-β1, M-siNC, or M-siTGF-β1 in the trachea with the frequency of two times a week. The body weight and mortality were recorded during the experimental period. The progression of the disease was monitored by daily tracking of the animal’s weight and survival rate, and the mRNA level expression of TGF-β1 was used as a successful criterion for the detection of BLM animal models. On the 28th day, mice were euthanized to collect lung tissue for subsequent experiments.

### Quantitative real time-PCR

Total RNA was isolated from cells and mice lungs utilizing trizol (Cat. No. R0016, Beyotime, China) as previously reported [50–52]. Briefly, this involved chloroform addition for RNA isolation, isopropanol precipitation, washing with 80% ethanol, air-drying, and resuspension in DEPC-treated water. The concentration of the extracted RNA was assessed using a Nanophotometer NP80 (Implen, Germany). Subsequently, For the synthesis of cDNA, the Evo M-MLV RT Kit (Accurate Biotechnology Co., Ltd) was utilized, adhering strictly to the manufacturer’s instructions. RT-qPCR analyses were conducted on CFX96™ Optics Module (BIO-RAD, USA) using qPCR Master Mix (Vazyme Biotech Co., Ltd). The relative expression levels of specific genes were calculated using the 2^−ΔΔCT^ method. The specific primers employed are detailed in Table 1.

**Table 1:** Primer sequences used for qRT-PCR

Gene name	Forward primer	Reverse primer
GAPDH	CATCACTGCCACCCAGAAGACTG	ATGCCAGTGAGCTTCCCGTTCAG
TGF-β1	TGATACGCCTGAGTGGCTGTCT	CACAAGAGCAGTGAGCGCTGAA
IL-6	TACCACTTCACAAGTCGGAGGC	CTGCAAGTGCATCATCGTTGTTC
CTGF	TGCGAAGCTGACCTGGAGGAAA	CCGCAGAACTTAGCCCTGTATG
Col I	TAAGGGTCCCCAATGGTGAGA	GGGTCCCTCGACTCCTACAT
Fn	CCCTATCTCTGATACCGTTGTCC	TGCCGCAACTACTGTGATTCGG
α-SMA	CCCAGACATCAGGGAGTAATGG	TCTATCGGATACTTCAGCGTCA
MMP2	CAAGGATGGACTCCTGGCACAT	TACTCGCCATCAGCGTTCCCAT
MMP9	GCTGACTACGATAAGGACGGCA	TAGTGGTGCAGGCAGAGTAGGA
E-cadherin	GGTCATCAGTGTGCTCACCTCT	GCTGTTGTGCTCAAGCCTTCAC
Vimentin	CGGAAAGTGGAATCCTTGCAGG	AGCAGTGAGGTCAGGCTTGGAA

*Abbreviations* GAPDH: glyceraldehyde-3-phosphate dehydrogenase; TGF-β1: transforming growth factor-β1; IL-6: interleukin-6; CTGF: connective tissue growth factor; Col I: collagen I; Fn: fibronectin; α-SMA: α-smooth muscle actin; MMP2: matrix metalloproteinase 2; MMP9: matrix metalloproteinase 9

### Western blotting analysis

Total protein was extracted from lysed lung tissue and cell samples through centrifugation, followed by separation via SDS-PAGE and transfer onto PVDF membranes (Merck Millipore, USA). A pre-incubation step in blot blocking buffer preceded the overnight incubation with primary antibodies against several biomarkers relevant to lung function and pathology, namely TGF-β1 (1:1000, Abcam, UK), E-cadherin (1:1000, Bioss, China), Vimentin (1:1000, ABclonal, USA), MMP2 (1:1000, ABclonal), MMP9 (1:1000, ABclonal), COL1A1 (1:1000, cell signaling Technology, USA), Fibronectin (1:3000, Abcam), α-Smooth Muscle Actin (1:1000, ABclonal), CTGF (1:1000, Abcam), SMAD2/3 (1:1000, ABclonal), and their phosphorylated counterparts, using GAPDH as a reference. The application of HRP-conjugated secondary antibodies and subsequent chemiluminescent detection provided the necessary visualization for analysis.

### Histology analysis

Processed through fixation in 4% paraformaldehyde, dehydration, paraffin embedding, and precision sectioning at 5 μm, lung tissue samples were prepared for histological examination. Sections were deparaffinized and stained with H&E, Masson’s trichrome, and Sirius red (Solarbio, Beijing, China), then examined with the OLYMPUS BX53M microscope (Tokyo, Japan) [67].

Lung tissue sections were dewaxed and hydrated and antigenically repaired at higher than 95 °C, followed by a sequence of treatments with H_2_O_2_, blocking solution, and primary antibodies at 4 °C for 16 h. The sections were further incubated with a biotinylated secondary antibody and SABC complex, culminating in chromogenic development with DAB.

### Hydroxyproline (HYP) content measurement

Hydroxyproline content within the samples, serving as an indicator of collagen tissue metabolism and fibrotic activity, was quantified using a hydroxyproline assay kit (Solarbio, Beijing, China) [68].

### Statistical analysis

Data analysis was performed using GraphPad Prism 8, with results presented as means ± standard error. Statistical significance was determined using ANOVA and Dunnett’s test for multiple comparisons, survival analysis via the log-rank test. Levels of statistical significance were denoted as *P < 0.05, **P < 0.01, ***P < 0.001.

## Results

### Preparation and characterization of M-siTGF-β1

M-Exos were isolated from milk, and specific exosome marker proteins (Tsg 101, CD63, and ALIX) were detected, indicating the successful purification of the MExos (Fig. 2A). First, siRNA was loaded into M-Exos using three different methods: electroporation, sonication, and the modified CaCl_2_ method. Next, the encapsulation efficiency (EE) of all these methods was measured. Among the three methods, the electroporation transfection method had the highest loading efficiency (Fig. S1A). Subsequently, the electroporation method was optimized by assessing the encapsulation rate under varying conditions. When other factors were kept constant and only the ratio of M-Exos to siRNA was changed (1:1, 1:5, 1:10, and 1:15), the highest encapsulation efficiency was exhibited at a ratio of 1:5 (Fig. S1B). Next, we investigated the encapsulation rate at various voltages (100 V, 160 V, 220 V, and 280 V) and found that the encapsulation rate increased with increasing voltage, reaching a maximum at 220 V and then decreasing (Fig. S1C). Therefore, an M-Exo to siRNA ratio of 1:5 and voltage of 220 V were selected as the optimal conditions for the electroporation transfection method.

Next, we loaded siRNAtargeted TGF-β1 to the M-Exos (M-siTGF-β1) using the electroporation transfection method, and the M-siTGF-β1 complex was nebulized using a nebulization needle. TEM revealed that the exosomes had a homogeneous morphology and a classic cup shape, independent of siRNA loading and nebulization (Fig. 2B). The average diameter of an M-Exo was approximately 100 nm. There was no difference in particle size before and after nebulization (Fig. 2C and D). Due to the negative charge of free siRNA, loading siRNA induced a change in the zeta potential of M-Exo. The absolute value of the zeta potential slightly decreased after nebulization (Fig. 2E). Moreover, the encapsulation efficiency of the siRNA-loaded M-Exos remained unchanged after nebulization (Fig. 2F). These results indicated that siRNA-loaded M-Exos maintain their integrity after nebulization. The stability of M-Exos in acidic environments was examined by incubating the M-Exos in PBS containing 10% FBS (pH 7.4 or 6.8) at 37 °C for 48 h and detecting any changes in particle size using NTA. The results indicated no significant changes in particle size, suggesting that M-Exos could maintain their structural integrity in the mildly acidic environment of lung tissue (Fig. 2G).

**Fig. 1:**
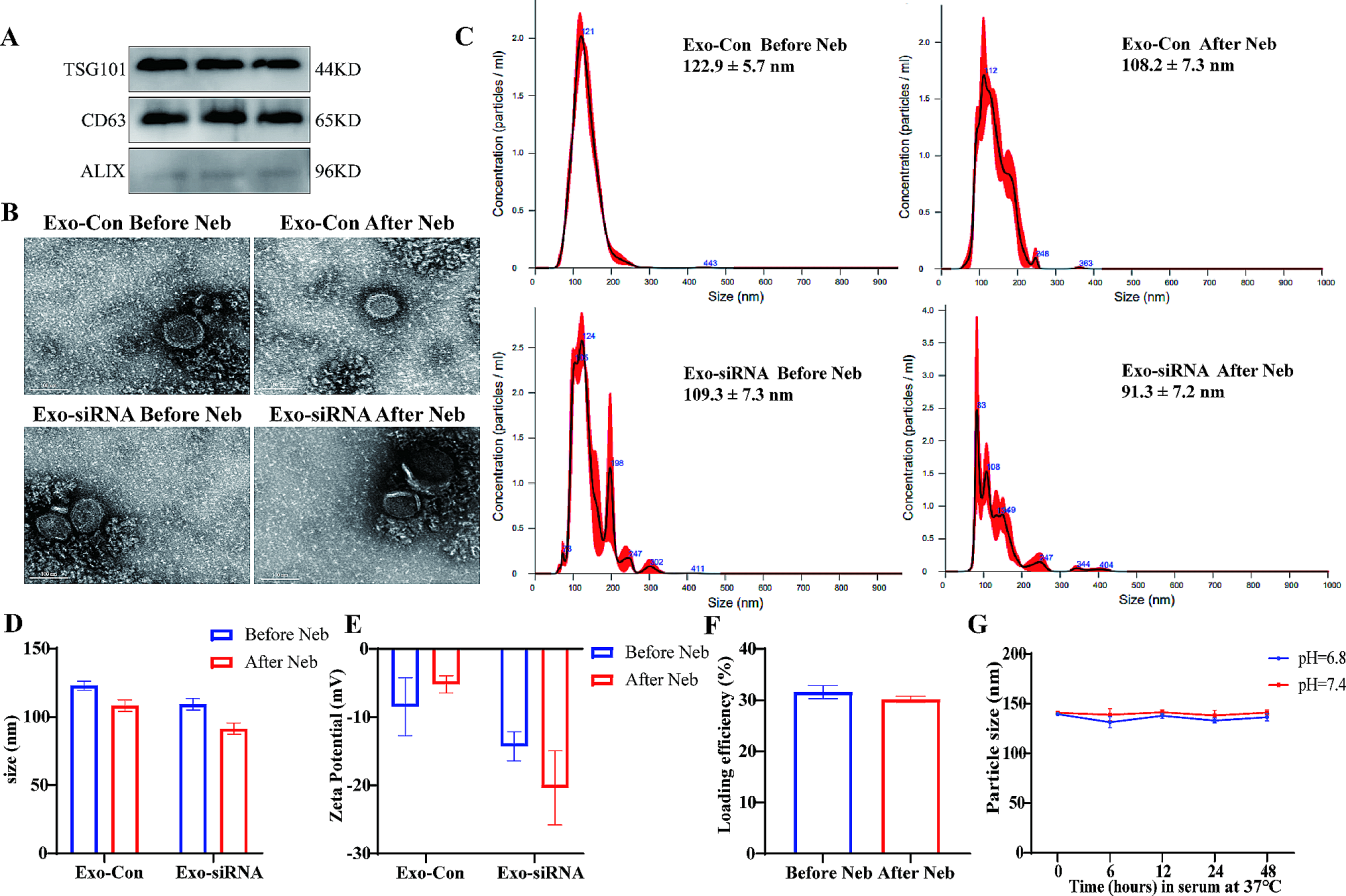
Characterization of siRNA-loaded M-Exos post-nebulization. (**A**) Expression of M-Exo signature protein. (**B**) TEM before and after M-Exo atomization. (**C**) Particle size before and after MExo atomization. (**D**) Changes in particle size. (**E**) Changes in zeta potential. (**F**) Changes in encapsulation rate. (**G**) Stability of M-Exo particle size (*N* = 3)

### Evaluation of collagen barrier penetration ability of exosomes in vitro

Exosomes, having the properties of lysosomal escape and collagen penetration, have the potential to serve as drug delivery systems [69, 70]. Exosomes protect small-molecule drugs from enzymatic or acidic degradation in lysosomes and ensure their effective delivery to cells at fibrotic sites [29]. To explore the lysosomal escape ability of M-Exos, Cy5-siRNA labeled M-Exos were applied to BEAS-2B cells, and green fluorescence labeling of lysosomes was performed to track the distribution of M-Exos in the cells at 3 h, 6 h, and 12 h. The results showed that M-Exos were co-localized with lysosomes at 3 h (Colocalization rate: 51.54 ± 9.08%), while at 6 and 12 h, a large number of Cy5-siRNA labeled M-Exos had escaped from the lysosomes, and only few M-Exos were co-localized with lysosomes (Colocalization rate: 30.96 ± 4.28% at 6 h and 29.37 ± 5.82% at 12 h) (Fig. 3A). The co-localization rates of M-Exos in lysosome based on the Pearson correlation coefficient showed that the co-localization decreased over time significantly (Fig. S2), indicating its good lysosomal escape ability. It might due to the membrane fusion between exosome and lysosome which could induce the release of cargo into cytoplasm quickly [71, 72].

To determine the collagen penetration capacity of the M-Exos, HFL1 cells underwent exposure to 1 µg/mL BLM for 24 h to facilitate its differentiation into myofibroblasts characterized by elevated α-SMA and collagen levels, indicative of collagen barrier formation, which was verified using western blotting (Fig. 3B and C). As can be seen from Fig. 3D, M-Exo could be taken up by HFL1 cells and the fluorescence intensity increased at 24 h compared with 12 h and 6 h. After the collagen barrier model was constructed, the fluorescence intensity of M-Exo taken up by HFL1 at 24 h did not differ from that of the normal group, suggesting that collagen formation does not have an effect on cellular uptake. Subsequently, DiR-stained M-Exos and M-Exos loaded with or without FAM-labeled siRNA were added into the BLM-induced HFL1 cells revealed a time-dependent uptick in M-Exo cellular uptake, notably higher at 24 h compared to 6 and 12 h. Moreover, unencapsulated siRNA exhibited poor cellular entry, while M-siTGF-β1 was captured by the cells, with the fluorescence intensity increasing over time (Fig. 3D). These results underscore the time-dependent proficiency of M-Exos in breaching the collagen barrier, thereby enhancing cellular uptake.

**Fig 2:**
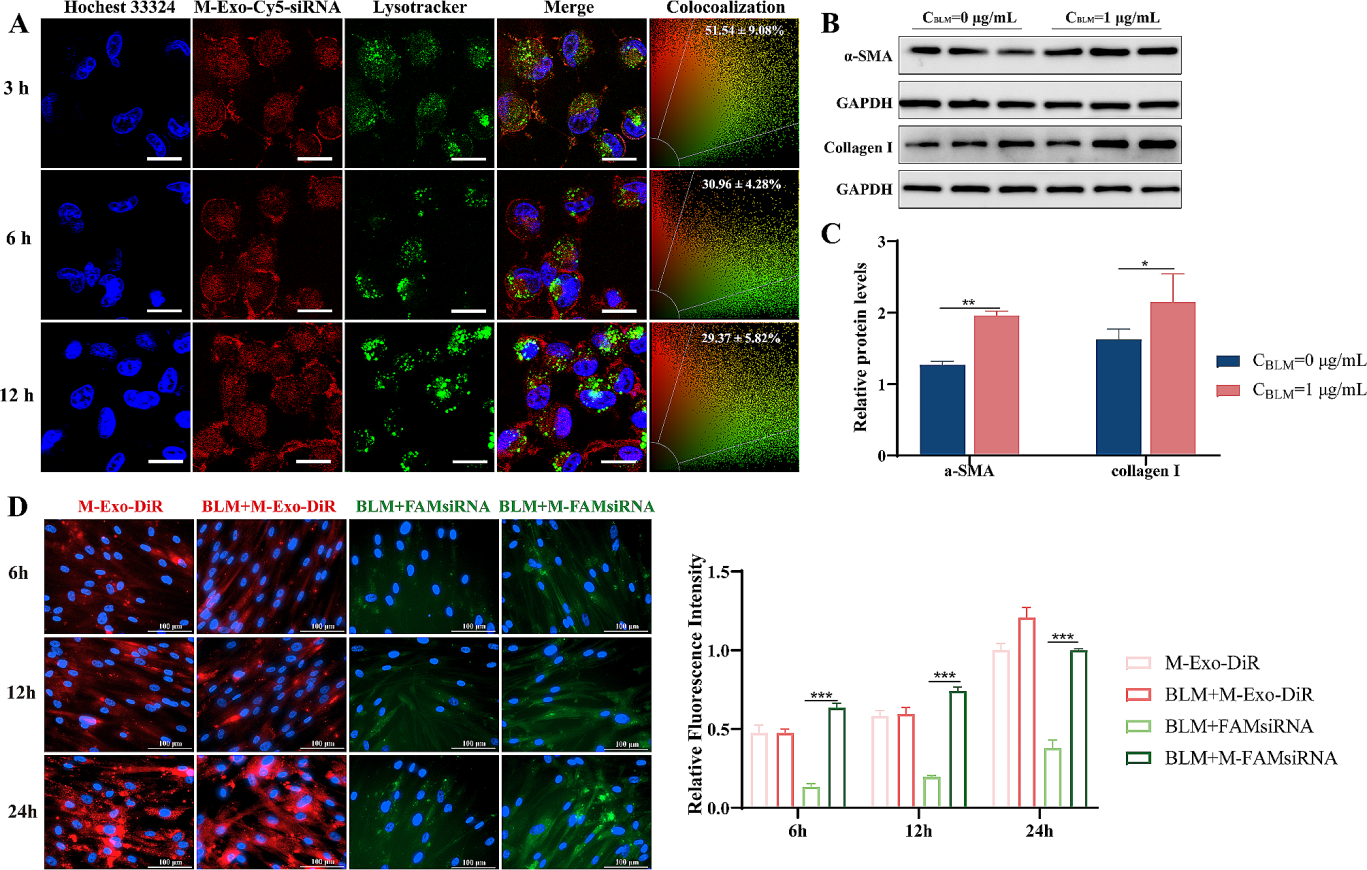
M-Exos can escape lysosomes and collagen penetration. (**A**) Colocalization images of M-Exos and lysosomes in BEAS-2B cells. Red, green, and blue fluorescence represent fluorescence of MExo, lysosome, nucleus after staining. The bar is 25 μm. (**B** and **C**) Protein expression of α-SMA and col I in BLM-treated HFL1 cells. (**D**) The ability of M-Exos to penetrate the collagen barrier in HFL1 cells. Blue fluorescence indicates DAPI-labeled nuclei. M-Exo-DiR indicates that M-Exos labeled with DiR were visible under red light. FAM siRNA indicates that FAM-labeled siRNA shows green light, and Merge indicates the overlap of two fluorescent signals at the same position in the cell

### M-siTGF-β1 alleviated cell migration and downregulated fibrosis factor expression in vitro

To analyz M-siTGF-β1’s role in counteracting BLM-evoked cellular injury in BEAS-2B pulmonary epithelial cells, we analyzed the ECM production of BEAS-2B cells treated with M-Exos, naked siRNA, siNC-loaded MExos (M-siNC), and siTGF-β1-loaded M-Exos (M-siTGF-β1). The upregulation of collagen I and fibronectin in cells challenged with bleomycin was significantly decreased after treatment with M-siTGF-β1, which was not seen in the other groups (Fig. 4A-C). To elucidate the role of M-siTGF-β1 in modulating the migratory behavior of BEAS-2B cells in response to bleomycin, a wound healing assay was employed. The data, illustrated in Fig. 4D-E, indicate a substantial decrease in the rate of wound healing in cells treated with M-siTGF-β1 at both 12-h and 24-h time points, relative to other groups.

**Fig. 3:**
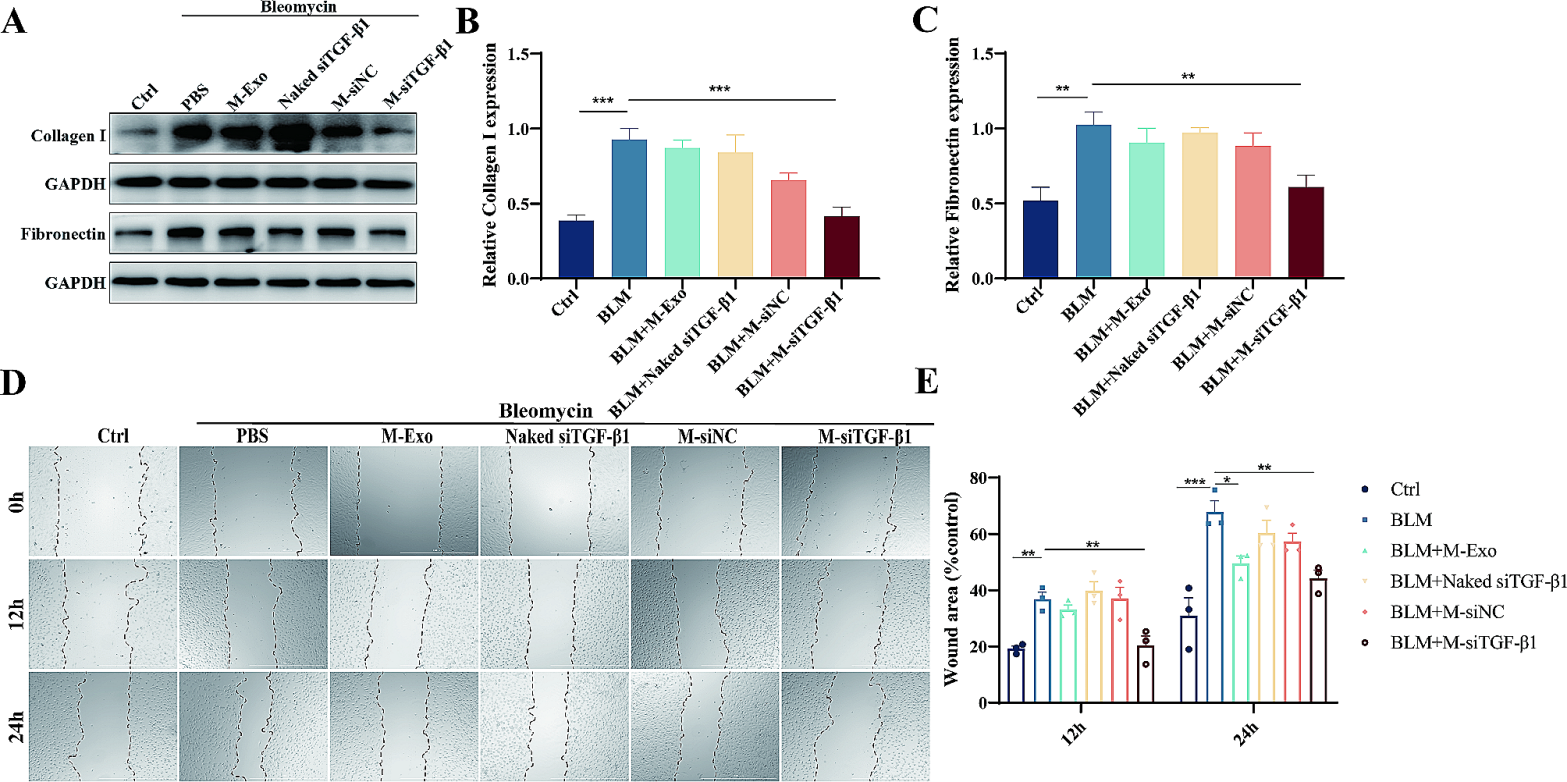
M-siTGF-β1 inhibit fibroblast activation and migration. (**A**–**C**) Western blotting of col I and fn in BEAS-2B cells treated with BLM. (**D** and **E**) Migration of BEAS-2B cells at different time points after BLM, BLM + M-Exo, BLM + Naked siTGF-β1, BLM + M-siNC, or BLM + M-siTGF-β1 treatment (*N* = 3)

### Biodistribution of DiR-labeled M-Exos after nebulization

The distribution of DiR-labeled M-Exos in the whole body of mice and dissociated organs was further investigated over a period of one week using an in vivo imaging system to determine the expression of the siRNA in the lungs after nebulization. The results showed that the vast majority of M-Exos resided in the lungs after nebulization, and the fluorescence signals gradually diminished after day 3 (Fig. 5A and B). To determine the initial point and frequency of drug administration, TGF-β1 expression was detected after treating mice with BLM. The results showed that TGF-β1 expression gradually increased and reached a peak on the 14th day; therefore, nebulized drug administration was initiated on the 14th day after BLM exposure (Fig. 5C). At 48 h after nebulized M-siTGF-β1 administration, reduced TGF-β1 expression was detected in mice with PF (Fig. 5D), indicating that M-siTGF-β1 was delivered to the lungs through tracheal nebulization and resulted in selective gene silencing.

**Fig. 4:**
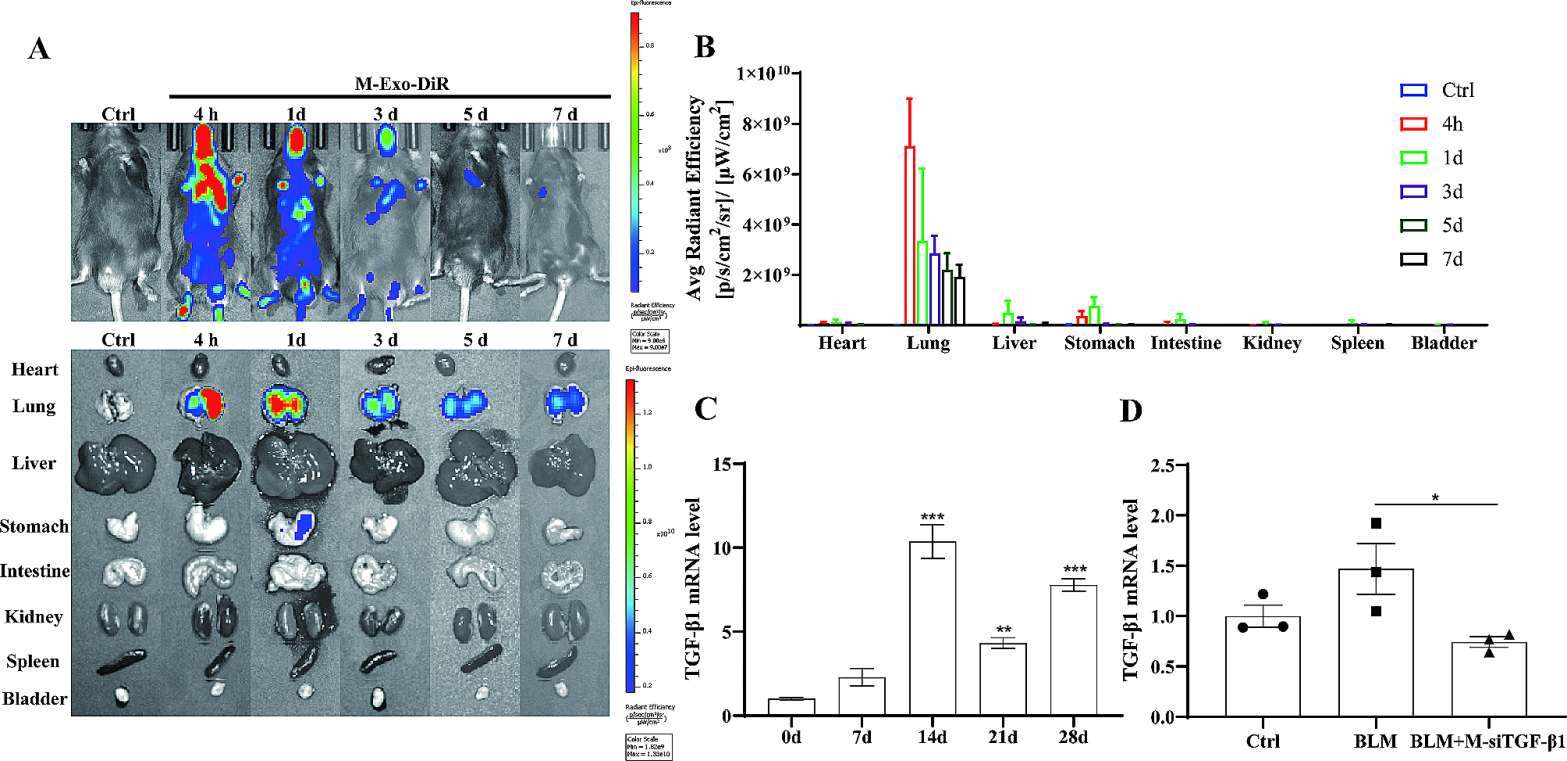
Biodistribution of M-Exos in the lungs after nebulization (**A**) in vivo imaging of the mice at 4 h and 1, 3, 5, and 7 days after DiR-labeled M-Exo nebulization. (**B**) quantitative analysis of fluorescence signals. (**C**) qPCR analysis the expression of TGF-β1 mRNA in the lung tissues of mice within 28 days. (**D**) qPCR analysis the expression of TGF-β1 mRNA in lung tissue 48 h after M-siTGF-β1 administration (*N* = 3)

### M-siTGF-β1 administration ameliorated BLM-induced lung inflammation

Mice were administered inhaled M-Exos, naked siRNA, M-siNC, or M-siTGF-β1 every 3 days after 14 days of BLM exposure to evaluate the mitigative effects of M-siTGF-β1 on inflammation. Lung tissues were harvested at day 28 as the experimental endpoint (Fig. 6A). The progression of the disease was monitored by daily tracking of the animals’ weight and survival rate. Weight monitoring revealed that M-Exos, naked siRNA, and M-siTGF-β1 treatment significantly alleviated BLM-induced body weight loss; however, the body weights of BLM-exposed mice were not restored to the weight of the control group (Fig. 6B). After the experiment, mice in the BLM + M-siTGF-β1 group had the least change in body weight compared with the initial body weight (Fig. 6C). In addition, the therapeutic application of M-siTGF-β1 significantly elevated survival probabilities among the BLM-challenged mice (Fig. 6D). Analysis of the histology showed that BLM toxicity directly caused severe congestive necrosis in both lungs, which was significantly improved in all therapeutic groups, especially in the M-siTGF-β1 group (Fig. 6E). Moreover, BLM administration notably increased the lung weight compared with that in the control mice, a condition that also improved after M-siTGF-β1 inhalation (Fig. 6F). At the pathological level, through H&E, Masson’s trichrome, and Sirius Red stains, disclosed severe lung tissue damage characterized by structural disarray, alveolar collapse, and augmented collagen deposition in the lungs of BLM-exposed mice. Conversely, M-siTGF-β1 treatment ameliorated lung histopathological damage and maintained normal lung morphology and hindered the accumulation of collagen caused by exposure to BLM (Fig. 6G-J).

qRT-PCR analysis of lung tissues elevated IL-6 mRNA expression levels in the BLM group when compared to the control group. Notably, this upregulation was significantly attenuated in the BLM group treated with M-siTGF-β1, as illustrated in Fig. 6K. Consistent with this, hydroxyproline (HYP) content, an indicator of collagen deposition, was considerably reduced in lung tissue homogenates of mice treated with M-siTGF-β1, in contrast to the BLM-only treated group, as depicted in Fig. 6L.

**Fig. 5:**
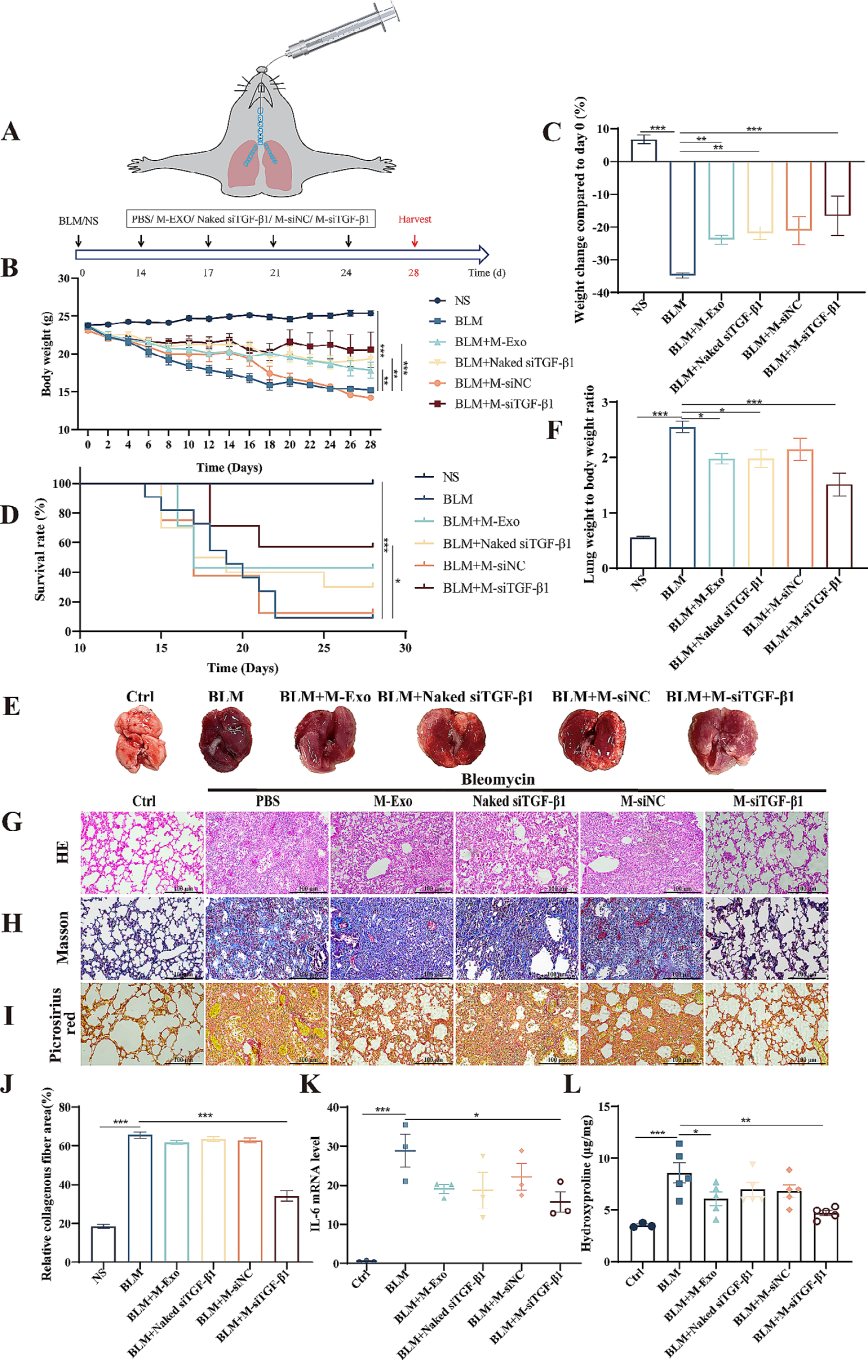
M-siTGF-β1 alleviated BLM-induced destruction of lung structure and inflammation (**A**) Experimental design of mouse PF model induced by BLM. (**B**) Body weight changes in mice throughout the experimental cycle. (**C**) Comparative body weight analysis from initial to the study’s endpoint. (**D**) Survival curves. (**E**) Lung tissue morphology of mice. (**F**) Assessment of changes in lung tissue weight. (**G**) Histopathological analysis using H&E staining. (**H** and **I**) Identification of fibrosis through Masson and Sirius Red staining. (**J**) Quantification of collagen area via Masson staining. (**K**) qPCR detection of inflammation-related factor IL-6 mRNA expression in lung tissue. (**L**) Hydroxyproline quantification offered a measure of total collagen content (*N* = 3–5)

### M-siTGF-β1 demonstrated antifibrosis effects on BLM-caused PF in mice

Excessive release of profibrotic and fibrotic factors leading to ECM deposition is characteristic of BLM-induced pulmonary fibrosis. Elevated levels of specific proteins, including pro-fibrotic enzymes MMP-2 and MMP-9, CTGF, and fibrotic markers collagen I, α-SMA, and Fn, have been implicated in the initiation and exacerbation of fibrotic conditions. Therefore, the effect of M-siTGF-β1 on the levels of these factors was explored. The qRT-PCR results demonstrated that following BLM administration, there was a significant rise in the transcriptional activity of fibrosis-related genes and ECM constituents, and a decrease following M-siTGF-β1 treatment (Fig. 7A-F). The antifibrotic impact of M-siTGF-β1 therapy on MMP-2, MMP-9, CTGF, collagen I, α-SMA, and Fn was further demonstrated through western blot analysis (Fig. 7G-J). Aligning with these findings, immunohistochemical assessments revealed a significant reduction in the expression of the myofibroblast marker α-SMA, as well as ECM components collagen I and Fn, in the lung tissues of mice treated with M-siTGF-β1 28 days post-bleomycin challenge, in comparison to the PBS, M-Exo, naked siTGF-β1, and M-siNC treated groups (Fig. 7K). This underscores the potent antifibrotic potential of M-siTGF-β1 in modulating key fibrotic processes.

**Fig. 6:**
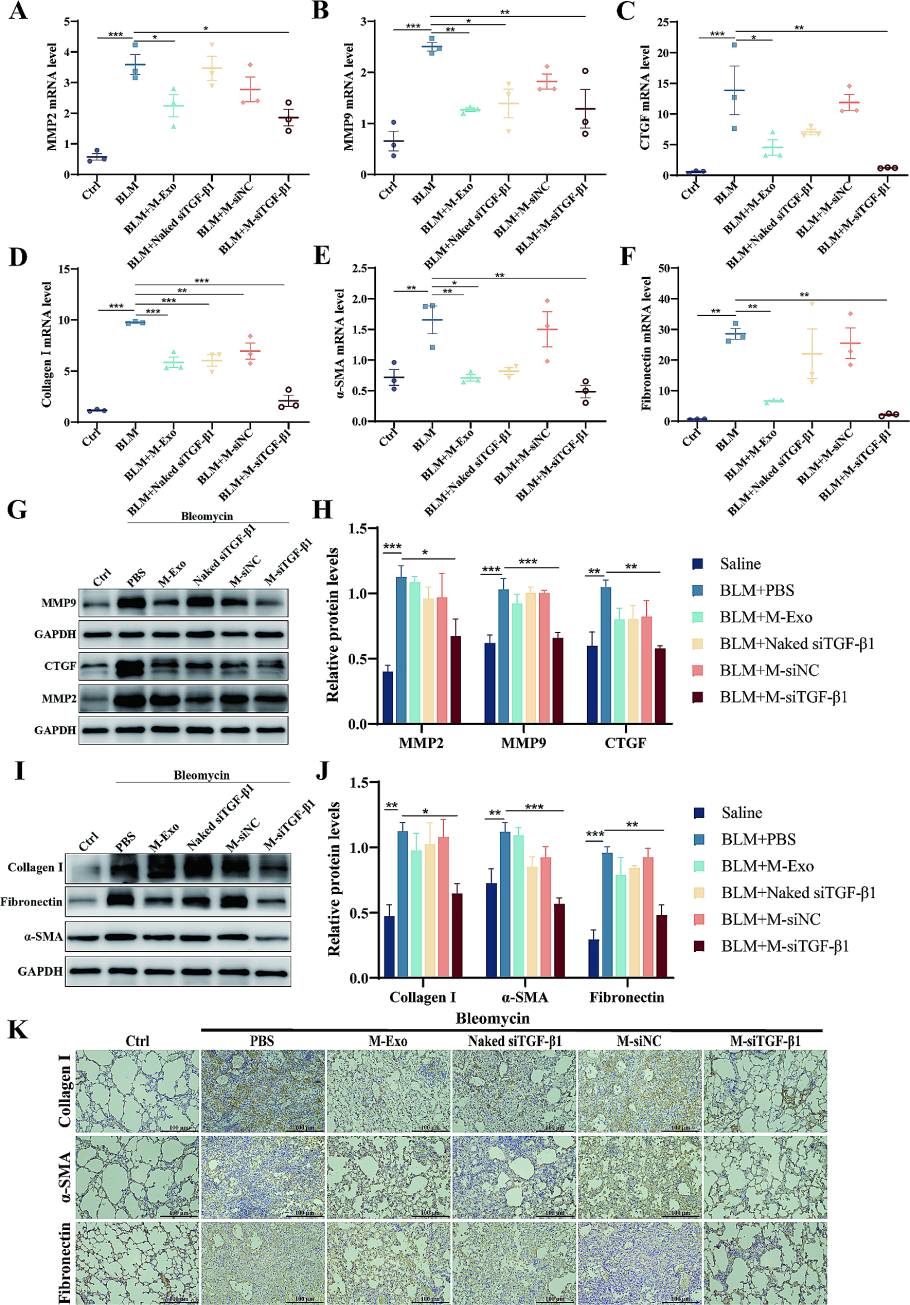
(**A**-**C**) qPCR detection of MMP2, MMP9, and CTGF mRNA expression in lung tissues. (**D**-**F**) qPCR detection of col I, α-SMA, and fn mRNA level in lung tissue. (**G** and **H**) Western blot detection of MMP2, MMP9, and CTGF protein expression in lung tissues. (**I** and **J**) Protein expression of col I, α-SMA, and fn in lung tissues. (**K**) Immunohistochemical staining for col I, α-SMA, and fn (N = 3-5)

### M-siTGF-β1-treatment alleviated BLM toxicity by inhibiting EMT via TGFβ/Smad2/3 signaling

To explore the mechanism underlying the effects of M-siTGF-β1 against BLM toxicity, we verified the siRNA-mediated knockdown efficiency of TGF-β1. This was followed by assessing the phosphorylation levels of Smad2/3 and quantifying the expression levels of pivotal EMT-associated molecules, both in cell culture and animal models. In the context of BLM-caused activation in BEAS-2B cells, a significant escalation in TGF-β1 levels and p-Smad2/3 was observed, delineating the fibrogenic response, which was substantially mitigated by M-siTGF-β1 intervention (Fig. 8A-C). Moreover, this therapeutic approach also led to a notable decrease in the mesenchymal marker vimentin and an increase in the epithelial hallmark E-cadherin, evidencing the reversal of EMT (Fig. 8D-F). Echoing the in vitro outcomes, the analysis of BLM-induced mice showed that after M-siTGF-β1 treatment, the level of TGF-β1 mRNA and protein was reduced due to RNA silencing, and the downstream phosphorylation level of Smad2/3 was subsequently reduced compared with that of the BLM-exposed mice (Fig. 8G-J). In addition, the mRNA (Fig. 8K and L) and protein level (Fig. 8M-O) of E-cadherin and vimentin were analyzed, revealing an augmentation in E-cadherin and a reduction in vimentin post-M-siTGF-β1 nebulization. Similar to the western blot results, the immunohistochemistry results demonstrated that silencing of the TGF-β1 siRNA gene restored E-cadherin levels and a decline in vimentin levels within the lung tissues (Fig. 8P). These results suggest that M-siTGF-β1 treatment protects against BLM toxicity and alleviated fibrosis in the lungs by impeding EMT through the TGF-β1-mediated Smad2/3 signaling route.

**Fig. 7:**
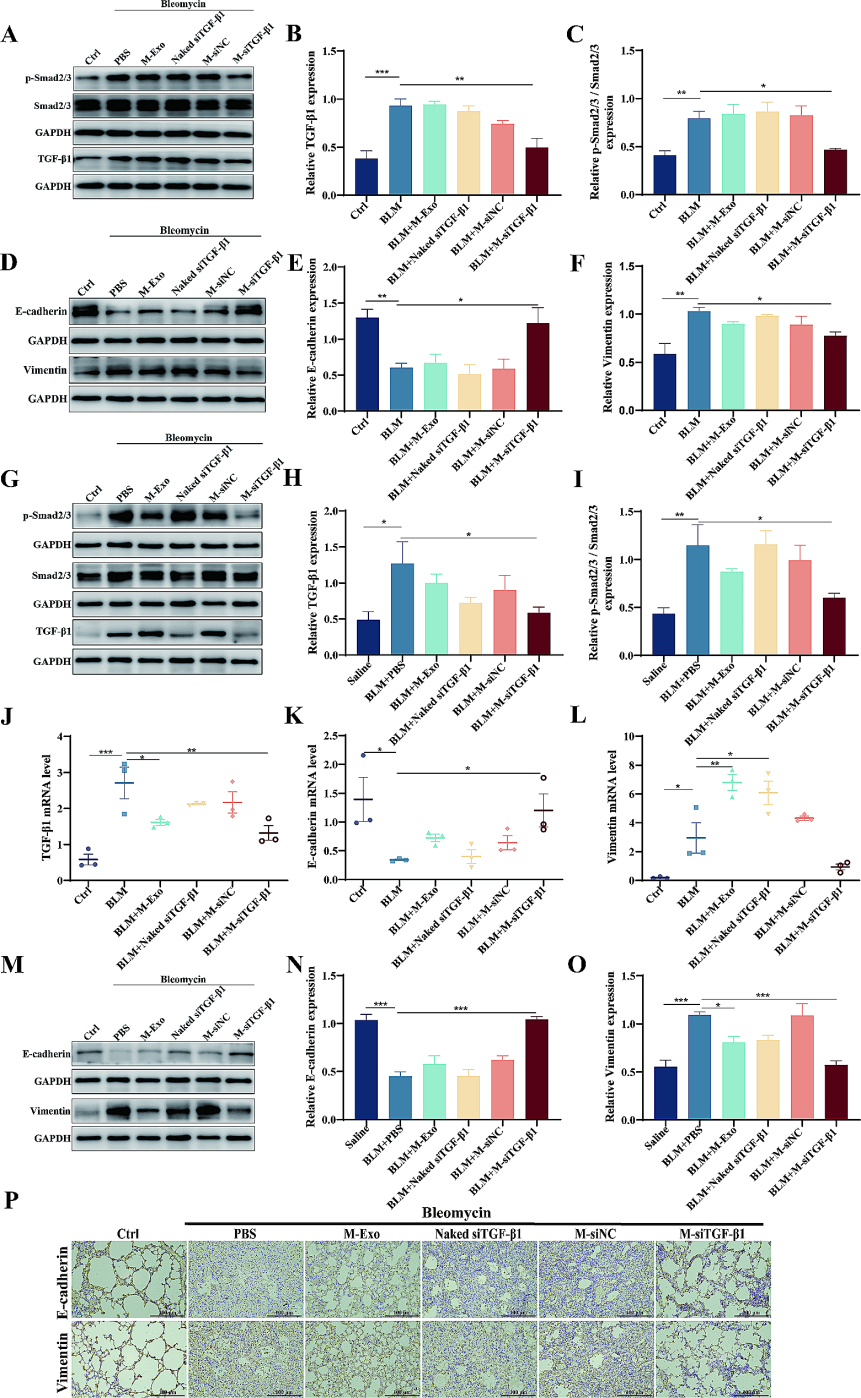
The M-siTGF-β1 mechanism protects against BLM-induced PF. (**A**-**C**) Protein expression of TGF-β1/Smad2/3 pathway in BEAS-2B. (**D**-**F**) E-cadherin and vimentin protein expression in BEAS-2B. (**G**-**I**) Protein expression of TGF-β1/Smad2/3 pathway in lung tissues. (**J**) TGF-β1 mRNA expression in lung tissues. (**K** and **L**) E-cadherin and vimentin mRNA expression by qPCR in lung tissues. (**M**-**O**) Protein expression of E-cadherin and vimentin in lung tissues. (**P**) Immunohistochemical staining for E-cadherin and vimentin (*N* = 3–5)

## Discussion

The exact etiology of PF development is unknown and requires further elucidation; however, it is known that fibrosis involves ongoing EMT, collagen deposition, and the overaccumulation of ECM proteins within the distal lung regions. These pathological changes invariably result in lung structure damage, impaired lung function, and eventual mortality. Given the pivotal role of TGF-β1 in PF pathophysiology [53], our research focused on targeting this growth factor to disrupt the TGF-β1 signaling cascade, thereby unveiling novel therapeutic strategies for PF management.

RNA interference (RNAi) can target certain mRNAs before they are synthesized and affect the protein levels implicated in disease processes. This feature renders RNAi a potentially effective therapeutic strategy to control the expression of disease-related genes, especially for diseases that are considered incurable by conventional methods [54–56]. Despite tremendous advances in in vivo drug delivery technology, targeting and delivering oligonucleotide therapies to sites beyond the liver remains a great challenge. Natural delivery vehicles have recently been increasingly recognized as an effective treatment agent for fibrotic diseases. Matsuda et al. invented a method to deliver siRNA from M-Exos loaded with β-connexin siRNA via liposomal solution. In contrast to M-Exos loaded with disordered siRNAs, M-Exos loaded with siRNAs showed significant knockout effects in vitro experiments [57]. Here we developed a M-Exos–based delivery platform to overcome the obstacles related to siRNA delivery targeting TGF-β1, and the developed nanoparticles were termed M-siTGF-β1. Combining the use of nanoparticle formulations with topical routes of administration can be seen as a viable solution to the problems posed by the many biological barriers that arise during gene delivery [58]. Nebulization emerges as a prominent, noninvasive technique for localized drug delivery in the treatment of lung diseases, distinguished by its painlessness and ease of use. In addition to being painless and relatively convenient, this approach offers numerous unique benefits, including enhanced drug deposition throughout the alveolar epithelium and bronchial, improved patient compliance and adherence, minimal systemic risk, suitability for repeat operations, and rapid onset of pharmacological action. The administration of siRNA therapies directly to the lungs through nebulized nanoparticle vehicles appears to be a viable and practical therapeutic approach. The experimental results showed that M-siTGF-β1 could withstand the shear force generated during nebulization, be taken up by target cells, avoid lysosome phagocytosis, and exert protective effect on siRNA.

In the realm of PF research, the Bleomycin-induced model of pulmonary fibrosis emerges as the quintessential therapeutic approach, underscored by its pervasive use in scientific investigations [59, 60]. BLM can be used to construct fibrosis models both in vivo and in vitro. The most significant major driver and mediator in the pulmonary fibrosis process is TGF-β1, which stimulates and attracts fibroblasts, accelerates EMT and triggers ECM synthesis [61]. The results of our initial experiments showed that the optimal stimulation time for BLM was 24 h. The optimal concentration of BLM for PF induction was determined to be 1 µg/mL from the TGFβ1 mRNA level and protein level.

In PF lung tissue, the most intuitive indicator is collagen deposition [62]. Fibronectin and α-SMA are effectors of myofibroblasts, which are essential for PF progression. Fibroblasts secrete ECM, which includes components such as fibronectin and interstitial collagen. These factors synthesize the initial scaffold, deposit key cells for tissue repair and regeneration, and recruit inflammatory factors [63]. Myofibroblasts are transformed from fibroblasts FMT and EMT action of epithelial cells. These fibroblasts and myofibroblasts respond to a variety of cytokines, including CTGF, IL-6, IL-13, and IL-33, results in their abnormal activation and the induction of key fibrotic pathways including TGF-β, Wnt, and Notch, which further contribute to PF, ultimately thickening the alveolar and bronchiolar walls and leading to remodeling of the lung tissue [64]. Our research, both in vivo and in vitro, has demonstrated that M-siTGF-β1 was capable of decrease BLM-induced inflammatory factor production and hydroxyproline content, inhibit the expression of fibrosis-associated molecules, and inhibit EMT formation stimulated by BLM-induced high TGF-β1 expression.

Smad2 and Smad3 are instrumental in TGF-β1’s role in driving fibrogenesis and the formation of the ECM. Upon TGF-β1 binding to its specific receptor, a cascade is triggered, phosphorylating Smad2 and Smad3. These phosphorylated forms amalgamate into a complex that migrates into the nucleus, constituting pivotal steps in the modulation of TGF-β1-driven gene expression and fibrotic disease progression [65, 66]. Research findings from both in vitro and in vivo settings indicate that M-siTGF-β1 attenuates the pronounced expression of p-Smad2/3 in cells and tissues afflicted with BLM-induced pulmonary fibrosis. However, there are limitations to this study, and it is crucial that TGF-β1 downregulation should not detrimentally impact other bodily organs. Further studies are warranted to address these concerns.

Although the results are encouraging, there is still room for improvement in the current work. Firstly, the process of pulmonary fibrosis is complex and lengthy, which involves associated mechanisms and intertwined pathways. Whether there are other signaling pathways besides the TGF-β1-mediated Smad2/3 signaling pathway can be further explored by mRNA sequencing of lung tissues from PF mice and screening for relevant differentially expressed genes. Secondly, some studies have reported that milk exosomes themselves have anti-inflammatory and anti-cancer effects. In studies using milk exosomes for drug delivery, milk exosomes alone showed significant inhibition of lung and breast cancer growth [39]. Further study of the effects of bovine milk exosomes on PF will help to further validate the effectiveness of the inhalable M-siTGF-β1 approach.

Constructing M-siTGF-β1 drug-carrying system with higher encapsulation efficiency and obtaining exosomes with high encapsulation rate has been a greater challenge. Obtaining exosome-siRNA drug-carrying systems with higher encapsulation efficiency through multiple drug-carrying methods is also the research goal of this study. Most of the intrapulmonary drug delivery in mice involved in previous studies was administered after incision, but the biggest adverse effect of this method is that: the animals’ self-control behavior is poor, and they are difficult to tolerate the surgical wounds, and are prone to scratching and gnawing the wounds, which affects the wound healing; meanwhile, the tracheotomy wound is large, with a high risk of infection and high mortality rate, which seriously affects the accuracy of the experimental results. Therefore, after reviewing a large number of literature and many experimental attempts, this project decided to use the mouse tracheal atomization drug delivery for the establishment of in vivo model, this method can directly hit the drug to the lungs, and can avoid the loss. Nucleic acid drug effectively reaches cellular subcellular organelles and so on to exert the drug effect, then it cannot be restricted by the cellular barrier in the transport pathway. The use of bovine milk exosomes as carriers can effectively avoid the degradation of gene drugs by enzymes or acids in vivo, and M-siTGF-β1 can reach the target organ more efficiently and thus exert its effects compared with bare siRNA.

## Conclusion

In this study, M-Exos were used as an RNAi delivery system, and siRNA-mediated low expression of TGF-β1 was introduced into exosomes by electroporation to form M-siTGF-β1 compounds. In vitro, M-siTGF-β1 affected the expression of EMT- and fibrosis-related molecules within BLM-challenged cellular model, while in vivo experiments revealed its capacity to mitigate PF symptoms. The therapeutic mechanism was thought to be exerted through the inhibition of inflammatory responses, EMT, and fibrosis-related molecules. MsiTGF-β1 inhibited the TGF-β1-mediated activation of Smad2/3, which may have affected the expression of EMT- and fibrosis-related molecules. MsiTGF-β1 could effectively deliver the target siRNA to the lungs, leading to selective gene silencing, has been shown to significantly improve survival outcomes in murine models of. This study provides a new way to target TGF-β1 silencing for the treatment of PF.

### Electronic supplementary material

Below is the link to the electronic supplementary material.

Supplementary Material 1

## Data Availability

No datasets were generated or analysed during the current study.
