# Supplementary material for: Nebulized milk exosomes loaded with siTGF-β1 ameliorate pulmonary fibrosis by inhibiting EMT pathway and enhancing collagen permeability
Source: J Nanobiotechnology. 2024 Jul 23;22:434. doi: 10.1186/s12951-024-02721-z (PMC11267965; doi:10.1186/s12951-024-02721-z)
Supplement: Supplementary file 1 — Supplementary Material 1 [file 12951_2024_2721_MOESM1_ESM.docx]

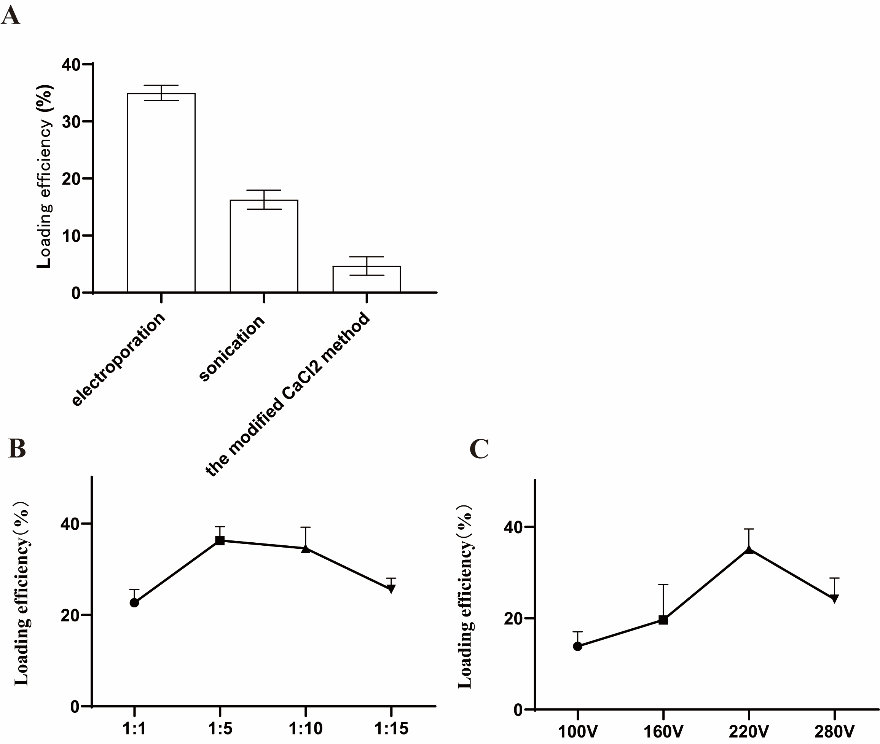


**Figure S1** **Examination of different loading methods and processes.**

(A) Encapsulation efficiency of M-EXO loaded siRNA by electroporation, sonication and the modified CaCl_2_ method. (B) Electroporation method to examine the encapsulation efficiency of different ratios of M-EXO and siRNA. (C) Electroporation method to examine the encapsulation efficiency under different voltages. (N=3)





**Figure S2** **Pearson's correlation coefficients of Lysosome and M-Exo fluorescence at 3, 6, and 12 h.**
